# Measuring the needs of dementia patients' caregivers: An assessment study from King Abdul-Aziz Medical City, Jeddah, Saudi Arabia

**DOI:** 10.12688/f1000research.129792.2

**Published:** 2023-11-22

**Authors:** Hani almugti, Hussain Alatram, Amani Alkhaldi, Abdulaziz Fadhel, Khalid Almalki, Khlood Aldhamen, Rokaya Alghamdi, Somaya Al Zhrani, Athbi Alenizi, Ahoud Altamimi, Faris Baawad, Waad Bin Afif, Ahmed Mkrshy, Amal Al Suliman, Arwa Alotaibi

**Affiliations:** 1 Primary Health Care Department, King Abdullah International Medical Research Centre, King Saud bin Abdul-Aziz University for Health Science, Jeddah, Western Region, Saudi Arabia; 2 Department of Emergency, Adan Hospital, Al-Ahmadi, Kuwait, Kuwait; 3 Nursing, Alhada Armed Forces Hospital, Taif, Saudi Arabia; 4 Faculty of Medicine, Umm Al-Qura University, Makkah, Saudi Arabia; 5 Pharmacy, Alnahdi Medical company, Taif, Saudi Arabia; 6 Dental Public Health, Safwa General Hospital, Qatif, Saudi Arabia; 7 Nursing, King Abdul-Aziz Medical City- National Guard, Jeddah, Saudi Arabia; 8 Nursing, AL-Mandag General Hospital, Baha, Saudi Arabia; 9 Faculty of Medicine, Jordan University of Science and Technology, Amman, Jordan; 10 Pharmacy, Serena Pharmacy, Qassim, Saudi Arabia; 11 Emergency Department, AlThagur General Hospital, Jeddah, Saudi Arabia; 12 Department of Medicine, Ministry of National Guard-Health Affairs, Jeddah, Saudi Arabia; 13 Laboratory, FARASAN GENERAL HOSPITAL, Jazan, Saudi Arabia; 14 Nursing College, Mohammad Al-Mana College for Medical Sciences, Dammam, Saudi Arabia; 15 Clinical pharmacy department for Intensive Care unit, East Arafat Hospital, Makkah, Saudi Arabia

**Keywords:** Caregiver, Saudi, Dementia, Alzheimer's disease, needs

## Abstract

**Background:**

Globally, dementia is estimated to become more prevalent as the population is aging. Patients with dementia are demanding on the long-term care from their caregivers. In order to maintain their well-being and minimize the impacts of long-term care, caregivers need comprehensive and supportive health services. This study aimed to improve and redesign the current healthcare service by assessing the needs of Saudi dementia patients’ caregivers using carers’ needs assessment for dementia (CNA-D).

**Methods:**

Through a cross-sectional design and convenient sampling technique (non-probability Sampling), this study was carried out in two Saudi home care centers. The caregivers who fulfilled the inclusion criteria (n=276) were enrolled in the study and completed the interview questionnaire. Data collection lasted for two months (September and October 2022). A Chi-square test was performed to determine the statistical significance between participants’ responses and their demographic data.

**Results:**

The majority of caregivers were females (76%). Their mean age was 38 years, ranging from 21 to 67 years. Two-thirds of caregivers spent more than one year on direct caregiving. About 60% of patients were male, and half were grandparents. Most caregivers (71%) did not live with their patients in the same household. Although caregivers rated all the addressed 13 needs in the present study as important, knowing more about the diseases was most important among caregivers of high education level. Further, long-term care gave caregivers more experience and reduced the need for practical support services.

**Conclusion:**

The results show that the principal caregiver in Saudi families were females, and a large proportion of dementia patients were males. There were variations in rating the importance of the addressed needs, which were associated with caregivers’ demographic characteristics. The findings of this survey demonstrate the importance of assessing the needs of family caregivers when developing social and healthcare services.

## Introduction

Dementia, including Alzheimer’s disease, is considered a public health challenge that impairs patients’ ability to maintain their daily living activities. ^1^ Because it is a progressive disorder affecting memory, cognitive abilities, and behavior, people living with this health condition need more support as they are unable to care for themselves. ^2^ Eventually, people living with Alzheimer’s will need round-the-clock care for months or even years in the advanced stage.

More than 55 million individuals worldwide are affected by dementia, making it one of the main factors that contribute to older people being disabled and dependent. ^3^ Given the steady rise in the percentage of people aged 65 and more, the global number of dementia cases is expected to reach 78 million in 2030 and 139 million in 2050. ^1^^,^^3^ In Saudi Arabia, the estimated number of people with Alzheimer’s disease is 130,000 cases, ^4^ and the number of Saudi older people (65 years and more) reached 854,281 people, representing 4.19% of the total Saudi population; their percentage is distributed by gender to 48.9% for males and 51.1% for females. ^5^

According to several studies, family members or friends are the principal caregivers for people with Alzheimer’s disease and related dementia. ^6^ Moreover, female caregivers provide care to more than two-thirds of people with Alzheimer’s disease and related dementia in their homes. ^7^ Compared with other older adults of different health conditions, the caregivers of people with Alzheimer’s disease and related dementia provide care for a considerably more extended period. Therefore, the demands of caregiving limit a caregiver’s ability to take care of themselves, leading to a poor quality of life and an increased risk of anxiety and depression. ^8^^,^^9^ Several studies have investigated the distress and burden of caregiving, and some studies described caregivers’ needs. ^2^^,^^7^ Family caregivers for newly diagnosed cases often have no care experience, feel unprepared, and need more support from the health care system to deliver adequate care.

There is a consensus on the importance of developing a national strategy to support caregivers. For instance, the Saudi Alzheimer’s Disease Association has recognized efforts to raise public awareness and direct decision-makers toward the financial, emotional, social, and health challenges that patients and caregivers face. ^10^ Despite these efforts, there is no agreement on what should be covered in needs assessments or how caregivers’ needs should be integrated into intervention and care planning.

The best assessment technique to measure the needs of dementia caregivers is a subject for debate. ^11^ Determining whether a particular need is met, under met, or unmet is considered a complementary component of assessing dementia caregivers’ needs.

Societies have different coping mechanisms originating from their cultural backgrounds and the available healthcare systems. ^12^ However, there is limited research covering the needs of family caregivers of dementia patients in Saudi society. This study aimed to support the caregiver’s role in the management plan of patients with dementia. The objective was to assess the Saudi caregivers’ needs using Carers’ Needs Assessment for Dementia (CNA-D) who were taking care of the registered dementia patients at the home care service of King Abdul-Aziz medical city in both cities Jeddah and Taif.

## Methods

### Ethical statement

After receiving approval from the ethical and scientific committees of King Abdullah International Medical Research Center at King Abdul-Aziz Medical City (approval number IRB/1767/22), the data-gathering procedure began and lasted for two months (September and October 2022). The anonymity of the data of the participants was maintained throughout the study. Written informed consent was obtained from participants, to participate in the current study.

### Study area and setting

This study was a cross-sectional study and was carried out in two cities, Jeddah and Taif, which are covered by the home care services of King Abdul-Aziz medical city. Home care services at King Abdul-Aziz medical city strive to provide health care assistance and nursing services for patients at their residences. Older adult patients are not the only target group for these services; there are other groups including patients with cancer, Alzheimer’s disease patients, and patients who had car accidents or head injuries resulting in a dementia diagnosis.

### Study subjects

The study recruited the dementia patients’ caregivers who met the following inclusion criteria: Saudi nationality with more than 18 years of age, who had direct close care with their patients for a minimum of six months.

### Sample size

Permission was granted from the home care department of King Abdul-Aziz Medical City to utilize their database, which consisted of a dementia patients list, including personal information about patients and their caregivers. Access to this database was only provided through permission from King Abdul-Aziz Medical City. However, it was used as a matrix to withdraw the sample through a convenient sampling technique.

The total number of dementia patients was 336 who had routinely scheduled health homecare visits and nursing services at their residences. After applying the inclusion criteria, we assumed that there would be one caregiver for each patient. All caregivers were invited to participate in the study remotely via mobile calls. Then, when we obtained their verbal approval to participate, we provided them with the consent form during the home care visits.

### Sampling technique

By a convenience sampling technique (non-probability Sampling) and during the home care visits, a team of three researchers conducted the interview questionnaire at the caregivers’ residences. The information technology (IT) section assisted in designing an electronic Arabic questionnaire integrated into our iPad to collect the data. All team members were trained and oriented to the objectives of this study. The team handled the following: explaining the aim of the study to the participants, obtaining informed consent from the participants, and answering the inquiries or questions concerning the questionnaire.

### Data collection methods, instrument used, and measurements

*Variables*

There were two variables included in this study: Dependent variables, where the needs of the caregivers of patients with dementia were considered; and independent variables, which included characteristics such as age, gender of caregivers, level of education, employment status, medical history of caregivers, relation to the patient, living situation, income, duration of giving care to patients, and stage of dementia (Alzheimer’s diseases).

*Needs of dementia patients’ caregivers*

Most dementia patients have family members who provide a different level of care and support. This kind of informal care is unpaid and always has characteristics of a particular scope of work, duration, and intensity if offered to older people with chronic comorbidities. Therefore, the caregivers are considered ‘secondary patients’ who deserve protection and guidance to become more competent and safe helpers for their loved ones. According to a prior systematic review, ^11^ no standard measure addresses all of the important needs of patients with dementia and their caregivers. Nevertheless, the Carer’s Needs Assessment for Dementia (CNA-D) is identified as a comprehensive tool for measuring the degree of severity and burden of the perceived needs in three main domains (Knowledge, practice, satisfaction with the health services and support). ^13^

*Questionnaire*

The questionnaire of this study was interview-administered and consisted of two sections as outlined The first section consisted of the following demographic data: caregivers’ characteristics including age, gender, level of education, relation to the patient, living situation, and duration of giving care to patients. It also included the patient’s characteristics for age, gender, and duration from diagnosis (less than one year- one year to two years- more than two years). The second section included the assessment of the caregivers’ needs for patients with dementia, which was adopted from the Carers’ Needs Assessment tool for Dementia (CNA-D) and the Family Inventory of Needs (FIN) questionnaire. ^14^ The CNA-D was developed and validated in 2005 and used to assess whether the required needs were addressed or not. We utilized these tools as a benchmark, it was validated, and we created a structural questionnaire from both of them in order to achieve our objectives. For the face and content validity, the questionnaire and related questions were translated into Arabic and then to English and then to Arabic again, and for revision, it was sent by email to the expert panel of specialists in family medicine, geriatric, community medicine, and mental health. The pilot study of 20 random participants was conducted in outpatient clinics of King Abdul-Aziz Medical City to measure the internal consistency (reliability) of the created structural questionnaire. The pilot also tested whether the questionnaire was comprehensible, clearly understood, and presented in a consistent manner. Patient information statements and consent forms were also tested for comprehension. The Cronbach alpha coefficient was 0.76, indicating good reliability.

The questionnaire contains 13 items, each rated on two subscales. The first subscale measured the importance of 13 care needs (the response options ranged from one (not important) to five (very important). The second subscale measured whether those needs rated as important have been met, partly met, or not met.

### Data management and analysis plan

The SPSS statistical software program for Windows was utilized for data entry and statistical analysis (version 20.0; IBM Corp., Armonk, NY, USA). Data entry and coding stages were performed for quality control purposes. Data were presented using frequencies and percentages for qualitative variables, and for quantitative variables, means and standard deviations were used. A Chi-square test was performed to compare variables among participants’ responses and their demographic data.

## Results

### Characteristics of the study subjects

Of the study population, 276 caregivers of dementia patients completed the questionnaire (response rate was 82%). Their mean age was 38 years, ranging from 21 to 67 years. Slightly more than two-thirds of the caregivers were female. Almost one-half of them had a level education of a bachelor’s degree (53%), and only 8% had a postgraduate degree. The most striking observation from the data was that most caregivers (71%) did not live in the same house as their patients ( Table 1).

**Table 1.** Demographic characteristics of the caregivers and their patients (n=276).

Demographic characteristics	Frequency	Percent (%)
**Caregivers’ characteristics**		
**Age**		
Range	21–67 years	
Median	36 years	
Mode	36	
Mean ± SD *	38 ± 8 years	
**Gender**		
Male	66	24
Female	210	76
**Level of education**		
Illiteracy	28	10
High school	80	29
Bachelor degree	146	53
Master/PhD	22	8
**Living situation**		
Same household	80	29
Different household	196	71
**Patients’ characteristics**		
**Age**		
Range	62–98 years	
Mean ± SD *	77 ± 11 years	
**Gender**		
Male	166	60
Female	110	40
**Duration of diagnosis with dementia**		
Less than two years	83	30
More than two years	193	70

* Standard deviation.

**Figure 1.**
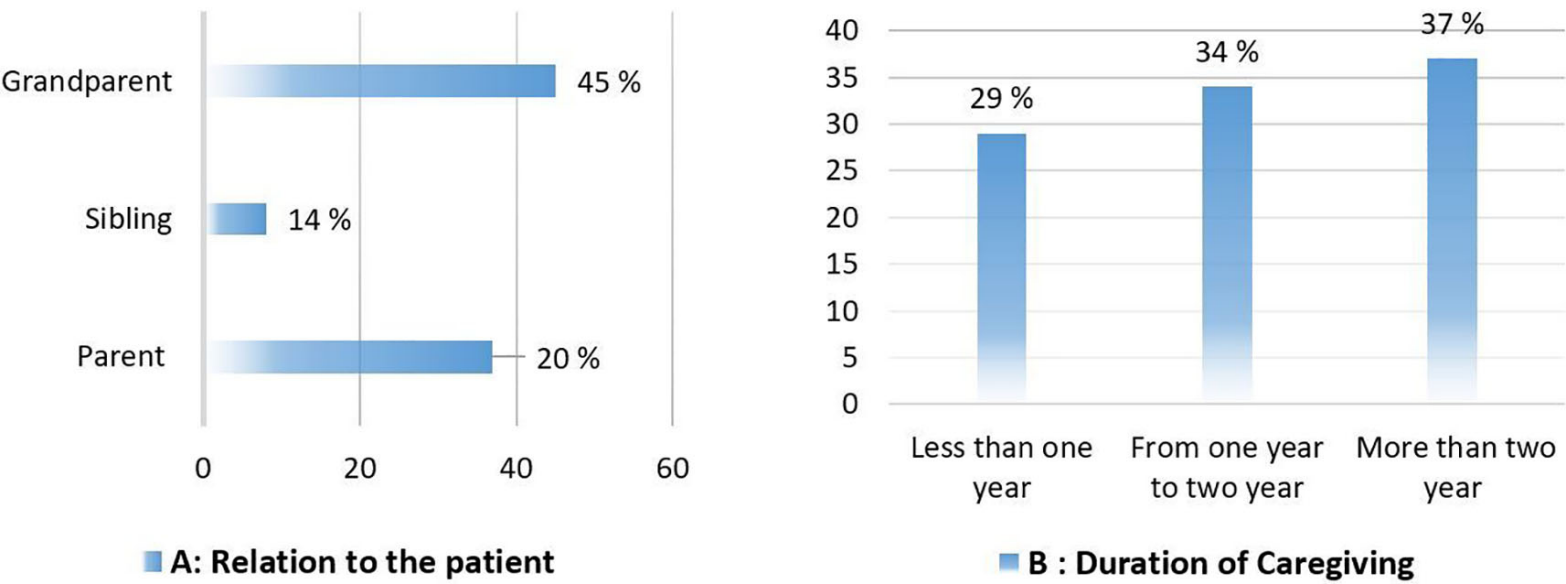
Caregivers’ percentages based on the patient’s relationship with them (A) and the duration of their caring (B).

Two-thirds of caregivers in the present study spent more than one year on direct caregiving. Almost half of the patients with dementia were grandparents ( Figure 1). Males constitute a large proportion of dementia patients (60%), and more than two-thirds have had dementia for more than two years ( Table 1).

### Important caregivers’ needs and unmet needs

Table 2 shows that all 13 needs were considered important by caregivers, and the majority (90%) indicated that knowing the exact outcome of dementia is important. However, 26% of them stated participation in group psycho-education was of low importance.

**Table 2.** Caregivers’ needs of patients with dementia (n=276).

Caregivers’ needs	Mean * ± SD **	Important needs % ***	Unmet needs %
1) Have my questions been answered honestly?	4.2 ± 1.4	82	8
2) Know specific facts concerning the patient’s prognosis	3.8 ± 1.3	82	13
3) Know the exact outcome of Dementia	4.1 ± 1.2	90	24
4) Know the symptoms that are caused by dementia or by the treatment used.	4 ± 1.3	87	11
5) Know the treatment plan	4 ± 1.3	84	13
6) Participation in group psycho-education for similar carers.	3.7 ± 1.5	74	34
7) Feel that the health professionals care about the patient? (Empathy)	3.8 ± 1.5	79	11
8) Know the names of health professionals involved in the patient’s care.	3.7 ± 1.4	76	18
9) Have information or training on practical skills for the carer (e.g., basic nursing skills) at home?	4 ± 1.3	79	24
10) Hotline where the carer can get advice in crises.	4 ± 1.4	79	32
11) Temporary help with the patient’s care at home.	3.8 ± 1.4	84	26
12) Have frequent home care visits.	3.7 ± 1.4	82	34
13) Care about my health status, be told about people who could help and support me with my problems.	3.6 ± 1.4	76	21

* Needs scale: 1 (not important) – 5 (very important).

** Standard Deviation.

*** Prevalence of important needs scored 4 (important) or 5 (very important).

Furthermore, almost a third of caregivers acknowledged that some needs were considered important but were not implemented, such as frequent home care visits, the availability of hotline services in emergencies, providing temporary assistance with the patient’s care at home, and receiving training in fundamental nursing skills at home.

### The relationship between the demographic characteristics and the four most important needs according to the participants’ answers

Regarding the association between the needs that most of the caregivers rated as important compared with other needs and their demographic characteristics ( Table 3), demonstrates a statistically significant difference between the older caregivers and the need to have help to take care of their patients. Moreover, the Chi-square test did not show significant differences between the gender of caregivers and their needs. Interestingly, the highly educated caregivers reported that knowledge about the diseases was important, compared with illiterate caregivers who indicated that assisting in caring was more important than knowledge.

**Table 3.** The relationship between the demographic characteristics and the four most important needs according to the participants’ answers.

	Is it important to know the exact outcome of dementia?		Is it important to know the symptoms caused by dementia or the treatment used?		Is it important to get temporary help with the patient’s care at home?		Is it important to have frequent home care visits	
	No	Yes	No	Yes	No	Yes	No	Yes
**Age group**								
○ 18–25 years	0	43	7	36	0	43	7	36
○ 26–35 years	8	79	8	79	8	79	8	79
○ 36–45 years	7	66	15	58	29	44	36	37
○ More than 45	15	58	7	66	7	66	0	73
***P value***	0.01 *		0.2		0.001 *		0.001 *	
**Gender**								
○ Male	4	62	7	59	6	60	8	58
○ Female	38	172	38	172	32	178	29	181
***P value***	0.1		0.7		0.06		0.88	
**Educational level**								
○ Illiteracy	14	14	7	21	0	28	0	28
○ High school	0	80	0	80	25	55	25	55
○ Bachelor degree	15	131	29	117	22	124	29	117
○ Master/PHD	0	22	0	22	7	15	7	15
***P value***	0.02 *		0.02 *		0.55		0.6	
**Relation to patient**								
○ Grandparent	11	141	11	141	32	120	43	109
○ Sibling	7	15	7	15	15	7	7	15
○ Parent	10	92	15	87	5	97	8	94
***P value***	0.01 *		0.02 *		0.001 *		0.02 *	
**Living situation**								
○ Same household	22	58	29	51	22	58	22	58
○ Different household	21	175	21	175	7	189	15	181
***P value***	0.08		0.01 *		0.002 *		0.02 *	
**Duration of caregiving**								
○ Less than one year	4	18	2	20	4	18	2	20
○ From one to two year	2	24	4	22	0	26	2	24
○ More than two years	2	26	4	24	8	20	10	18
***P value***	0.4		0.8		0.01 *		0.012 *	

* Statistically significant at p < 0.05.

Furthermore, Table 3 illustrates that according to caregivers, dementia patients who were grandparents and did not live in the same house require more assistance in caring from the healthcare system. However, providing temporary help with the patient’s care at home and having more frequent home care visits to caregivers was not reported as important by almost a third of caregivers in the present study if the length of caregiving was more than two years.

## Discussion

This cross-sectional study was conducted to assess the needs of the caregivers who were taking care of the registered dementia patients at the home care program of King Abdul-Aziz medical city Jeddah in order to ensure that the reported important needs from their perspective were met or not, thus will assist in improving the health service and promoting the health for both the patient and their caregivers.

The results of this study show the mean age of patients with dementia was 77 years, and slightly more than half of them were male. This supports the data from previous studies ^15^ that stated that dementia is more common in the older adults, and with advancing age, the risk may reach up to 17-fold. ^16^ On the other hand, the gender differences in dementia incidence varied in the medical literature, ^15^ and there was no difference in risks reported between males and females. ^17^ However, other studies ^15^^,^^17^ indicated that dementia is more common in women than men, with no specific gender differences among patients of dementia after 90 years of age.

The current study found that the mean age of caregivers was 38 years, and the majority of them were female. These demographic features were similar to those of earlier studies, ^7^^,^^18^ emphasizing that in many societies and cultures, women are the principal caregivers for their family members who need care and are always available to support and assist them. Furthermore, although two-thirds of the caregivers in the present study did not live with their patients in the same house, they dreaded the patient’s admission to a nursing home because in Saudi society, caring for patients with dementia in their homes is a social and religious value.

Concerning the caregiver’s educational level, the present study’s results showed that the great majority of the participants had at least a high school degree, and more than 80% of them rated the need for additional knowledge and practical support as important. Generally, the educational level is considered a challenge in providing an adequate health service. These findings support the idea that ^19^ for designing an efficient health program, it is vital to assess the education level of the targeted population to enhance the accessibility to those who need them most. Following this conclusion, service providers should reassess whether their services are being utilized effectively or not from the perspective of their clients.

Prior research ^20^ indicated that although there is always a caregiver’s need, the type of needs may vary depending on the phase of dementia and how long the patient has had dementia. At the beginning of the disease, knowledge about its nature, causes, symptoms, and treatment methods are considered important needs that help to understand the behavioral changes that occur in their patients. ^20^^,^^21^ As the disease progresses, the need becomes more important for practical support (including daily personal care, eating, and cleaning). ^21^ This finding aligns with the results of the present study that found that the longer the period of patient care, the more the caregiver has the experience, and the need for practical support services decreases. However, a previous study ^22^ highlighted that caregivers with long care periods are prone to loneliness and isolation from their social environment. Therefore, at this stage, they may need more intense psychological support.

Finally, a number of important limitations need to be considered. First, the generalizability of these results is restricted due to the small numbers of patients and their caregivers. Second, these findings are limited by using a cross-sectional design, and longitudinal studies are recommended to provide a clearer view of how informal carers’ needs and service utilization change throughout the disease. Third, our data did not address how frequently the needs should be offered in every stage of the disease. Despite these limitations, this study provides insight into caregivers’ needs that should be assessed regularly and considered for improvement.

## Conclusion

Dementia is a progressive disorder affecting older people who need more support as they are unable to care for themselves. The results of the present study show that the principal caregiver in Saudi families were females, and males represented a large proportion of dementia patients. Although caregivers rated all the addressed 13 needs in this study important, knowledge about the diseases was important among caregivers of a high level of education compared with practical help, which was more important than knowledge among low-educated caregivers. In addition, this study has shown that experience in long-term care decreases the need for practical support services. From the caregivers’ perspective, there were variations in the importance rating of the needs. However, assessing the demographic characteristics of the caregivers and measuring the important needs from their perspective are considered the backbones of designing a cost-effective health program for such a challenging public health problem.

## Data Availability

The datasets generated and analyzed during the current study are not publicly available because access to the public-use dataset must be granted by the ethical committee from the King Abdullah International Medical Research Center at King Abdul-Aziz Medical City. Retention of original data is a legal obligation for the principal investigator of this study - breach of confidentiality may have legal consequences for participants of the survey. Researchers who ask for confidential data can obtain access authorization to data from the King Abdullah International Medical Research Center at King Abdul-Aziz Medical City ( https://kaimrc.ksau-hs.edu.sa/). Zenodo: Questionnaire of Research article titled (Measuring the needs of dementia patients’ caregivers: An assessment study from King Abdul-Aziz Medical City, Jeddah, Saudi Arabia). https://doi.org/10.5281/zenodo.7533431. ^23^ This project contains the following extended data:
• Arabic questionnier.docx (Blank Arabic copy of the questionnaire used in this study). • Questionnier 7.docx (Blank English copy of the questionnaire used in this study). Data are available under the terms of the Creative Commons Zero “No rights reserved” data waiver (CC0 1.0 Public domain dedication).
