# Supplementary material for: Measuring the needs of dementia patients' caregivers: An assessment study from King Abdul-Aziz Medical City, Jeddah, Saudi Arabia
Source: F1000Res. 2023 Nov 22;12:296. Originally published 2023 Mar 17. [Version 2] doi: 10.12688/f1000research.129792.2 (PMC11997542; doi:10.12688/f1000research.129792.2)
Supplement: Peer review history

# Reviewer report: Reviewer response for version 2

**DOI:** 10.5256/f1000research.159128.r373389

**Published:** 2025-04-14

**Authors:** Christine J. Jensen

**Affiliations:** 1 Riverside Center for Excellence in Aging and Lifelong Health, Williamsburg, USA

I appreciated being able to see the other reviewers' feedback which were in line with mine.

 I would like to suggest the author changes reference from the "dementia patient" to the "patient with dementia" or "patient living with dementia."

 There are also several places where the authors assume that caregiving always leads to poor quality of life. This should be tempered with "can lead" or "may lead to poorer quality of life." Some caregivers experience satisfaction and reward yet we sometimes fail to ask them.

 I appreciated the observation about caregivers with more education and that their identified needs are different than those with less education. This is an important distinction that would serve those who provide home care well to know.

 I agreed with the reviewer who indicated there really aren't independent variables as we don't have control over these in the sample. Simply referring to them as variables is adequate.

Is the work clearly and accurately presented and does it cite the current literature?

Yes

If applicable, is the statistical analysis and its interpretation appropriate?

Yes

Are all the source data underlying the results available to ensure full reproducibility?

Yes

Is the study design appropriate and is the work technically sound?

Yes

Are the conclusions drawn adequately supported by the results?

Yes

Are sufficient details of methods and analysis provided to allow replication by others?

Yes

Reviewer Expertise:

Dementia care, training for caregivers, both family and professional, community-based care supports for older adults

I confirm that I have read this submission and believe that I have an appropriate level of expertise to confirm that it is of an acceptable scientific standard.

# Reviewer report: Reviewer response for version 2

**DOI:** 10.5256/f1000research.159128.r224754

**Published:** 2023-11-30

**Authors:** Peter George Tian

**Affiliations:** 1 Division of Care of the Elderly, Department of Family Medicine, University of Alberta, Edmonton, AB, Canada

No further comments.

Is the work clearly and accurately presented and does it cite the current literature?

Yes

If applicable, is the statistical analysis and its interpretation appropriate?

Yes

Are all the source data underlying the results available to ensure full reproducibility?

No

Is the study design appropriate and is the work technically sound?

Yes

Are the conclusions drawn adequately supported by the results?

Partly

Are sufficient details of methods and analysis provided to allow replication by others?

Partly

Reviewer Expertise:

I'm a Research Director in the Care of the Elderly, with degrees in medicine, clinical epidemiology, and health sciences education.

I confirm that I have read this submission and believe that I have an appropriate level of expertise to confirm that it is of an acceptable scientific standard.

# Reviewer report: Reviewer response for version 1

**DOI:** 10.5256/f1000research.142504.r217175

**Published:** 2023-11-15

**Authors:** Sheria Robinson-Lane

**Affiliations:** 1 Department of Systems, Populations, and Leadership, University of Michigan, Ann Arbor, Michigan, USA

More work on cultural variances in caregiver needs is necessary. This work adds to that literature. Consider the following suggestions:

1. An additional close review to correct minor grammatical mistakes would be beneficial.
2. Please note that “elderly” is no longer considered an appropriate term for older adults within current age-inclusive language guidelines. Just use “older adults,” and where appropriate specify the age range you are referencing.
3. Sampling technique: please clarify and specify what is meant by built into our iPad. Was a link accessed that allowed the completion of an electronic survey that was built into Qualtrics, RedCap or a similar program? 
4. Results: Confidence intervals should be reported alongside p values.
5. Conclusion: I don’t see findings that indicate the physical, psychological, or mental health outcomes of the study participants. Consider removing the sentence that references this and focus the conclusions on reported findings.

Is the work clearly and accurately presented and does it cite the current literature?

Yes

If applicable, is the statistical analysis and its interpretation appropriate?

Yes

Are all the source data underlying the results available to ensure full reproducibility?

Yes

Is the study design appropriate and is the work technically sound?

Yes

Are the conclusions drawn adequately supported by the results?

Partly

Are sufficient details of methods and analysis provided to allow replication by others?

Partly

Reviewer Expertise:

Family Caregiving

I confirm that I have read this submission and believe that I have an appropriate level of expertise to confirm that it is of an acceptable scientific standard, however I have significant reservations, as outlined above.

# Response

**Published:** 2023-11-19

**Authors:** Hani Al-mugti

**Affiliations:** Primary Health Care Department, King Abdullah International Medical Research Centre, Saudi Arabia

1) An additional close review to correct minor grammatical mistakes would be beneficial.

- Thank you, it is considered.

2) Please note that “elderly” is no longer considered an appropriate term for older adults within current age-inclusive language guidelines. Just use “older adults,” and where appropriate specify the age range you are referencing.

- Thank you for this information. To be honest, I do not know this. However, we changed the elderly to older adults.

3) Sampling technique: please clarify and specify what is meant by built into our iPad. Was a link accessed that allowed the completion of an electronic survey that was built into Qualtrics, RedCap or a similar program? 

- The information technology (IT) section in King Abdul-Aziz Medical City assisted in designing an electronic Arabic questionnaire integrated into our iPad to collect the data.

4) Results: Confidence intervals should be reported alongside p values.

- I understand that our table would be more valuable with the confidence interval. Nonetheless, in our result, mentioning the participant number was more indicative and clarified the existence of statistical significance, if any.

5) Conclusion: I don’t see findings that indicate the study participants' physical, psychological, or mental health outcomes. Consider removing the sentence that references this and focus the conclusions on reported findings.

- Accepted.

# Reviewer report: Reviewer response for version 1

**DOI:** 10.5256/f1000research.142504.r168746

**Published:** 2023-04-26

**Authors:** Peter George Tian

**Affiliations:** 1 Division of Care of the Elderly, Department of Family Medicine, University of Alberta, Edmonton, AB, Canada

Thank you for sharing your study. Indeed, the needs of caregivers is an important research topic. Here are some comments/suggestions to improve the manuscript you shared.

1. Abstract. “This study aimed to improve and redesign the current healthcare service by assessing the needs of Saudi dementia patients’ caregivers using carers’ needs assessment for dementia (CNA-D).”
  Comments/Suggestions: I suggest deleting the reference to improving/redesigning healthcare service. Although the study results may be used in service improvement/redesign, the study itself had nothing to do with healthcare service improvement/redesign.
2. “People living with Alzheimer’s will need…”
  Comments/Suggestions: I suggest using Alzheimer’s Disease rather than just Alzheimer’s.
3. “By a convenient sampling technique (non-probability Sampling)…”
  Comments/Suggestions: I suggest using “convenience sampling” instead of “convenient sampling.” Describe how you did your convenience sampling (e.g., Does 276 represent all those who consented from the 336 eligible patients? Were there caregivers who consented but you did not include?) 
4. “There were two variables included in this study: Dependent variables…and independent variables…”
  Comments/Suggestions: Since this is a cross-sectional study, there are no independent and dependent variables, no cause-and-effect implications. I suggest rephrasing this sentence to state that the study had many variables (more than 2). 
5. “The second section included the assessment of the caregivers’ needs for patients with dementia, which was adopted from the Carer’s Need Assessment tool for Dementia (CNA-D) and Family Inventory of Needs (FIN) questionnaire.”
  Comments/Suggestions: Did you secure permission to adopt the CNA-D and FIN questionnaire? How was the CNA-D and FIN adopted? Aside from translation, did you use all the original questions? Did you modify or delete some questions? If you modified some questions, how were the questions modified?
6. “The Cronbach alpha coefficient was 0.76…”
  Comments/Suggestions: Describe how the Cronbach’s alpha coefficient was derived? Was this coefficient for the answers to CNA-D, FIN, or both? How does your computed coefficient compare with the Cronbach alpha coefficients of the original CNA-D and FIN?
7. “A Chi-square test was performed to determine the statistical significance between participants’ responses and their demographic data.”
  Comments/Suggestions: I suggest deleting the words “statistical significance.” The Chi-square was used to compare variables among participants.
8. “Their mean age was 38 years…”
  Comments/Suggestions: I suggest adding standard deviation.
9. Comments/Suggestions: Table 2 shows the mean and SD of questions or statements. The table’s legend shows the need scale. Kindly include this scale in the section about the questionnaire.
10. Comments/Suggestions: Table 2 shows Unmet needs %; yet, there is no footnote for this. How was this derived? This may need to be described in the section about the questionnaire.
11. Comments/Suggestions: I suggest revising Table 3 to show which two percentages were being compared for each p value stated. For example, for the question “Is it important to know the exact outcome of dementia” and the age group, the p value is 0.01; which 2 percentages were compared by the chi-squared test?
12. Comments/Suggestions: I suggest toning down the conclusion to statements consistent with the study results.

Is the work clearly and accurately presented and does it cite the current literature?

Yes

If applicable, is the statistical analysis and its interpretation appropriate?

Yes

Are all the source data underlying the results available to ensure full reproducibility?

No

Is the study design appropriate and is the work technically sound?

Yes

Are the conclusions drawn adequately supported by the results?

Partly

Are sufficient details of methods and analysis provided to allow replication by others?

Partly

Reviewer Expertise:

I'm a Research Coordinator in the Care of the Elderly, with degrees in medicine and clinical epidemiology.

I confirm that I have read this submission and believe that I have an appropriate level of expertise to confirm that it is of an acceptable scientific standard, however I have significant reservations, as outlined above.

# Response

**Published:** 2023-04-27

**Authors:** Hani Al-mugti

**Affiliations:** Primary Health Care Department, King Abdullah International Medical Research Centre, Saudi Arabia

I would like to thank you for your valid input, and I believe this will make our work better.

 Here are our responses to your comments:

**1) I suggest deleting the reference to improving/redesigning healthcare services. Although the study results may be used in service improvement/redesign, the study itself had nothing to do with healthcare service improvement/redesign**.

- Thank you for this point. As you know, generally, any care service provided by a health institute is considered a healthcare service. In our study, needs assessment was our approach to improve the health care service for patients and their caregivers. 

**2) I suggest using Alzheimer’s Disease rather than just Alzheimer’s.**

- Accepted

**3) By a convenient sampling technique (non-probability Sampling)…”** **I suggest using “convenience sampling” instead of “convenient sampling.”**

- Accepted

**Describe how you did your convenience sampling (e.g., Does 276 represent all those who consented from the 336 eligible patients?**

- We included the caregivers who agreed to participate and met our inclusion criteria.

**Were there caregivers who consented but you did not include?)**

- No

**4) Since this is a cross-sectional study, there are no independent and dependent variables, no cause-and-effect implications. I suggest rephrasing this sentence to state that the study had many variables (more than 2).**

- Agree, from cross-sectional studies, we used to formulate a hypothesis. However, (independent and dependent variables) are statistical terms that explain and identify the association between variables, and It is not only dedicated to experimental or other analytic studies.

**5) Did you secure permission to adopt the CNA-D and FIN questionnaire?**

- Yes

**How was the CNA-D and FIN adopted? Aside from translation, did you use all the original questions?**

- For the face and content validity, the questionnaire and related questions were translated into Arabic and then to English and then to Arabic again, and for revision, it was sent by emails to an expert panel of specialists in family medicine, geriatric, community medicine, and mental health.

**Did you modify or delete some questions? If you modified some questions, how were the questions modified?**

- Modification was based on expert opinions and that was through face validation and content validation of the experts

**6) The Cronbach alpha coefficient was 0.76…”**

**Describe how the Cronbach’s alpha coefficient was derived?**

- After the pilot study, the Cronbach's alpha is derived by first calculating the correlation coefficient between each pair of items in the questionnaire or test. Then, the average of all of these correlation coefficients is calculated, and this value is adjusted based on the number of items in the questionnaire or test and the average inter-item correlation.

**was this coefficient for the answers to CNA-D, FIN, or both?**

- Both

**How does your computed coefficient compare with the Cronbach alpha coefficients of the original CNA-D and FIN?**

- We didn’t

**7) I suggest deleting the words “statistical significance.” The Chi-square was used to compare variables among participants.**

- Accepted

**8) “Their mean age was 38 years…”**

**Comments/Suggestions: I suggest adding a standard deviation.**

- Already presented in the table

**9) Table 2 shows the mean and SD of questions or statements. The table’s legend shows the needed scale. Kindly include this scale in the section about the questionnaire.**

- Already mentioned in the Questionnaire section (last paragraph)

**10) Table 2 shows the Unmet needs %; yet, there is no footnote for this. How was this derived? This may need to be described in the section about the questionnaire.**

- Already mentioned in the Questionnaire section (last paragraph)

**11) I suggest revising Table 3 to show which two percentages were being compared for each p value stated. For example, for the question “Is it important to know the exact outcome of dementia” and the age group, the p value is 0.01; which 2 percentages were compared by the chi-squared test?**

- We prefer to keep it without percentages

**12) I suggest toning down the conclusion to statements consistent with the study results.**

- Thank you
